# Combining methylated RNF180 and SFRP2 plasma biomarkers for noninvasive diagnosis of gastric cancer

**DOI:** 10.1016/j.tranon.2024.102190

**Published:** 2024-11-13

**Authors:** Zhihao Dai, Jin Jiang, Qianping Chen, Minghua Bai, Quanquan Sun, Yanru Feng, Dong Liu, Dong Wang, Tong Zhang, Liang Han, Litheng Ng, Jun Zheng, Hao Zou, Wei Mao, Ji Zhu

**Affiliations:** a School of Public Health, Nanjing Medical University, Nanjing, 211166, China; b Department of Radiation Oncology, Zhejiang Cancer Hospital, Hangzhou, 310000, Zhejiang, China; c Hangzhou Institute of Medicine (HIM), Chinese Academy of Sciences, Hangzhou 310000, China; d Department of Oncology, Affiliated Hospital of Jiaxing University, The First Hospital of Jiaxing, Jiaxing, 31400, China; e Hebei University of Engineering, Handan, 056009, China; f Virtue Diagnostics Co., Ltd

**Keywords:** Gastric cancer, Diagnostic biomarker, DNA methylation, Ring finger protein 180, Secreted frizzled related protein 2

## Abstract

**Introduction:**

Gastric cancer (GC) is a common malignant tumor, and early diagnosis significantly improves patient survival rates. This study aimed to investigate the diagnostic value of ring finger protein 180 (*RNF180*) and secreted frizzled protein 2 (*SFRP2*) in GC.

**Materials & Methods:**

A total of 165 healthy individuals, 34 patients with precancerous gastric lesions, and 104 patients with confirmed GC were divided into training and validation sets; methylated *RNF180* and *SFRP2* were detected in circulating DNA from blood samples. Six models, including those based on logistic regression, Naive Bayes, K-nearest neighbor algorithm, glmnet, neural network, and random forest (RF) were built and validated. Area under the curve (AUC), sensitivity, specificity, positive predictive value, and negative predictive value were determined.

**Results:**

In the training set, the RF model with *RNF180* and *SFRP2* (R + S) had an AUC of 0.839 (95 % CI: 0.727–0.951), sensitivity of 60.3 %, and specificity of 85.5 % for diagnosing GC. The RF model with R + S+ Tumor markers had an AUC of 0.849 (95 % CI: 0.717–0.981), sensitivity of 62.8 %, and specificity of 87.1 %. In the validation set, the RF model with R + S had an AUC of 0.844 (95 % CI: 0.774–0.923), sensitivity of 87.8 %, and specificity of 69.2 %. The RF model with R + S + Tumor markers had an AUC of 0.858 (95 % CI: 0.781–0.939), sensitivity of 85.4 %, and specificity of 76.9 %.

**Conclusion:**

Our results suggest that *RNF180* and *SFRP2* could serve as diagnostic biomarkers for GC when using the RF model.

## Highlights

• Early diagnosis of gastric cancer (GC) dramatically improves survival rates.
• We studied the diagnostic value of RNF180 and SFRP2 in early diagnosis of GC.
• We built, trained, and validated six diagnostic models.
• Using RNF180 and SFRP2 within a random forest model was the most effective.
• RNF180 and SFRP2 could serve as diagnostic biomarkers for GC using the RF model.

## Introduction

Gastric cancer (GC) is the third leading cause of cancer incidence and mortality worldwide (4.8 % incidence and 6.8 % mortality). In China, Itis the fifth leading cause of cancer incidence and the third most common cause of mortality (7.4 % incidence and 10.1 % mortality) (https://gco.iarc.fr/en, 2024 (accessed 8 July 2024)). Early detection, diagnosis, and treatment are effective methods to reduce cancer incidence and mortality. Currently, emerging treatments for gastric cancer are targeted therapy and immunotherapy [1,2]. The therapeutic efficacy, immune-related adverse events (irAEs), and prognosis significantly affect patient survival [[3], [4], [5], [6], [7]]. Although substantial progress has been made in the treatment of GC, further research and development is needed. Improving early detection, reducing recurrence, and optimizing therapeutic strategies are major challenges and prospects for GC management. Biomarkers can be used not only as predictors of treatment efficacy but also as predictors of irAEs for gastric cancer diagnosis [[8], [9], [10]]. However, traditional strategies for early diagnosis and screening of GC rely on serum tumor markers, imaging, and endoscopy, including carcinoembryonic antigen (*CEA*), *CA19–9, CA125*, and other [[11], [12], [13]]. Traditional methods such as CT, ultrasound, positron emission tomography-computed tomography (PET-CT), and MRI have limited effectiveness in detecting early GC [[14], [15], [16]]. Although gastroscopy is highly sensitive and specific, it is invasive and inconvenient, and it may lead to infection [17]. Tumor markers, widely used in clinical practice, lack sufficient sensitivity and specificity [18]. Existing studies have shown that the areas under the curve (AUCs) of *CEA, CA19–9,* and *CA125* for diagnosing GC are only 0.625, 0.577, and 0.585, respectively, and their combined AUC is 0.776 [19,20]. Therefore, there is an urgent need to develop accurate and effective techniques for the early diagnosis and screening of GC in clinical practice.

Aberrant gene methylation is a common molecular event in tumorigenesis and development, occurring in the early stages of cancer and serving as a valuable diagnostic biomarker [21]. The approximately 30 million specific methylation sites (CpG sites) in the human genome provide abundant signals for cancer detection [22]. Efficient bisulfite sequencing and machine learning can be used to read methylated DNA sequences and identify those that are aberrantly methylated, while analysis of methylated regions can reveal cancer-specific methylation patterns in early screening tests [23,24]. Additionally, the unique DNA methylation patterns of cancer cells in vivo can be used to identify cancer locations [25,26]. DNA methylation profiles are more diverse than typical mutation signals, offering greater sensitivity and broader cancer coverage, leading to improved diagnostic efficiency and wider clinical applications [[27], [28], [29]]. Given the extensive differences in DNA methylation patterns between normal and cancer cells, previous studies have focused on aberrant methylation in the promoter regions of one or a few candidate genes. Detection techniques for early cancer diagnosis have been developed, and pan-cancer diagnostic methods, such as circulating free DNA (cfDNA) methylation detection, are now entering international clinical validation stages [[30], [31], [32], [33]].

Ring finger protein 180 (*RNF180*) is a newly discovered member of the ring finger protein family, located on the long arm of chromosome 5, which expresses E3 ubiquitin ligase activity [34]. RNF180 is involved in various physiological processes, including cell growth, differentiation, and tumorigenesis Several studies have shown that *RNF180* is silenced or downregulated in a variety of cancers, including gastric [35], colorectal [36], and ovarian cancers [37]. Furthermore, *RNF180* has demonstrated potential for non-invasive diagnosis of GC. Researchers conducted a meta-analysis of nine studies and found that the combined sensitivity of *RNF180* for diagnosing GC was 54.0 %, while the specificity was 80.0 %[38]. It was reported that the AUC for diagnosing GC using *RNF180* was 0.723 (95 % CI: 0.694–0.752), with a sensitivity of 42.6 % and specificity of 87.3 %[39].

The WNT signaling protein plays multiple roles in tumorigenesis. Secreted frizzled related proteins (SFRPs), a family of five secreted glycoproteins, are potential negative regulators of the WNT signaling pathway [40]. The *SFRP2* gene encodes one of these proteins, which plays an important role in regulating cell growth, apoptosis, and differentiation [41]. Additionally, *SFRP2* has been identified as a candidate tumor suppressor gene and a major contributor to tumorigenicity in colorectal [42], esophageal [43], and GC [44], where it is often silenced by promoter hypermethylation. It was reported that *SFRP2* is frequently hypermethylated in the early stages of colorectal cancer [42]. It has been shown that *SFRP2* has diagnostic potential for GC. It was reported that the sensitivity of methylated *SFRP2* for detecting GC and gastrointestinal epithelial hyperplasia was 60.9 % and 56.3 %, respectively, while the specificity was 86.0 %[45]. Although the sensitivity and specificity of *RNF180* and *SFRP2* for diagnosing GC alone were low, their combination yielded better results, with a sensitivity of 82.4 % and a specificity of 42.86 % [46].

Therefore, we explored the sensitivity and specificity of combining *RNF180* and *SFRP2* with traditional tumor markers for detecting GC. Additionally, we compared six models to determine the best one for diagnosing GC using *RNF180* and *SFRP2*. This study provides valuable insights for improving the screening and diagnosis of GC.

## Materials and methods

### Patient recruitment

We selected 303 fresh plasma specimens that were collected from February 2023 to February 2024 at Zhejiang Cancer Hospital, including 165 healthy cases, 34 cases of gastric precancerous lesions, and 104 cases of GC. All patients met the following criteria: (1) age 40 years or older; (2) recent gastroscopy and tumor marker examination; (3) Blood collection volume of approximately 10 mL; (4) complete case information. Clinical and pathological information of the study subjects included sex, age, screening date, and TNM stage. The study was approved by the Ethics Committee of Zhejiang Cancer Hospital, and all participants provided informed consent (approval number: GC-IVD-2022–01).

### Sample collection, processing, and storage

Peripheral blood samples (10 mL) were collected using single-use vacuum blood collection tubes from Xiamen Zhixuan Biologicals, and we performed methylation determination of the *RNF180* and *SFRP2* genes. The blood samples were processed within 30 min of collection or stored at 2–8 °C for up to 8 h before being processed into plasma. Blood samples were initially centrifuged at 1350±150 rcf for 12 min, and the plasma was transferred to clean 15-mL centrifuge tubes. The plasma was then centrifuged again at 1350±150 rcf for 12 min, and the upper layer of plasma was transferred to a new centrifuge tube. The prepared plasma samples were stored in a refrigerator below −70 °C for long-term storage.

### cfDNA extraction and bisulfite conversion

Plasma free DNA was extracted using the Magnetic Bead Method Free DNA Bulk Extraction Kit (Guangzhou Meiji, IVD5435), and all eluted cfDNA products were subjected to bisulfite conversion. The extracted cfDNA was processed using EZ-96 DNA Methylation-Gold™ MagPrep (Zymo, D5042). Finally, the processed cfDNA was subjected to fluorescent polymerase chain reaction (PCR) detection using primer probes for recognition of methylated and unmethylated sequences (PCR model: SLAN96P).

### Determination of RNF180 and SFRP2 gene methylation

We qualitatively assessed *RNF180* and *SFRP2* gene methylation using the Digestive Tumor Multi-Gene Methylation Detection Kit (Virtue Diagnostics Co., Ltd.). The PCR conditions were 95 °C for 15 s, 56 °C for 30 s, and 60 °C for 30 s to collect fluorescence, for a total of 40 cycles. We used ACTB (β-actin) as an internal control to assess the DNA input amount and the validity of PCR amplification. Results were considered valid if the ACTB Ct was ≤45 and the external negative and positive controls met the validity criteria specified by the manufacturer. Ct values for RNF180 and SFRP2 were also recorded to enhance the detection of the reagents using different algorithms.

### Detection of serum tumor markers

We detected *CEA, CA199,* and *CA125* using the electrochemiluminescence method of the Department of Clinical Laboratory of Zhejiang Cancer Hospital, utilizing Siemens Centaur XP-3 and Abbott ALINIQ analyzers. Values of *CEA* ≥ 5.0 ng/mL, *CA199* ≥ 37.0 U/mL, and *CA125* ≥ 35.0 U/mL were considered positive.

### Cell lines and cultures

Two gastric cancer cell lines (MKN-7 and HGC-27), and the human normal gastric mucosa cell line GES-1 were cultured in RPMI-1640 medium (Sigma) supplemented with 10 % fetal bovine serum (Gibco) and incubated at 37 °C with 5 % CO2.

### Cell transfection and qPCR

Small interfering RNA (siRNA) transfection GC cells were transfected with thenoxal-specific siRNA at a final concentration of 20 μM using Lipo2000 following the manufacturer's instructions. Scrambled siRNA served as a negative control. Transfection efficiency was assessed using qPCR after the transfection. The target sequences of siRNA were as follows: siRNF180: 5′-ACCAUGAUGCCAGGUCCCUAA TT-3′ and siSFRP2: 5′-CCGAAAGGGACCTGAAGAAAT-3′.Total RNA was extracted using RNA Easy Fast Tissue/Cell Kit (TIANGEN, DP451). Real-time quantitative PCR (qPCR) was performed using SYBR qPCR premix (Tiangen), and signals were detected with the SLAN96P qPCR system (Biosystems). All qPCR reactions were performed in at least three biologically independent replicates. The relevant primers (forward and reverse sequences) for qPCR are listed in Supplementary Table S1.

### Cell count kit 8 (CCK-8) assay

The CCK8 assay was performed according to the manufacturer's instructions (Beyotime Biotechnology). Briefly, 100 µL of cultured cells (2000 cells) were added to each well of a 96-well plate and incubated for 1 to 6 days. At each time point, 10 µL of sterile CCK-8 was added to each well and incubated at 37 °C for 2 hours. At the end of the incubation, the absorbance (optical density, OD) of each well was measured at 450 nm using a microplate reader.

### Statistical analysis

Statistical analysis was performed using SPSS Version 26 and GraphPad Prism version 9.1. Independent samples *t*-test was used for comparative analysis between the two groups, and mDNA and mRNA levels were analyzed using one-way ANOVA. Statistical significance was set at p < 0.05. The training set samples were grouped into subgroups to apply different classification algorithms, including random forest (RF), Naive Bayes (NB), k-nearest neighbor (KNN), neural network (NNET), glmnet, and logistic regression (LR). The results of the reagents to detect each target and other test results were combined, and models with diagnostic value were selected by analyzing AUC. The best models screened in the training set were used to validate the subject work characterization curves (ROC) in the validation set. The AUC, sensitivity, specificity, positive predictive value (PPV), and negative predictive value (NPV) were calculated. For undetermined samples, we set the Ct value to 40 (the maximum number of PCR cycles).

## Results

### Levels of *mRNF180, mSFRP2, CEA, CA125,* and *CA199* in different groups

**Fig. 1:**
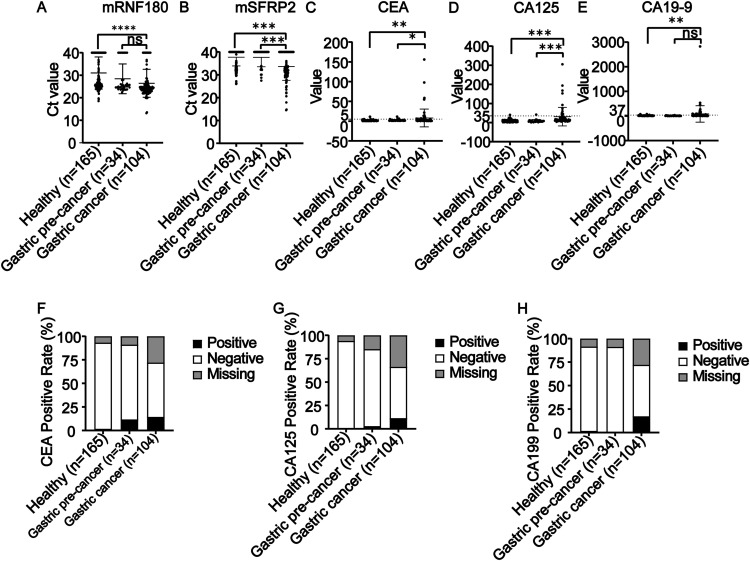
RNG180, SFRP2 methylation and tumor marker levels in each group. (A, B) Ct values for RNF180 and SFRP2. (C, D, E) CEA, CA125, CA199 measurements. DNA methylation levels and conventional tumor marker measurements were shown for gastric cancer, gastric cancer precancerous lesions, and healthy subjects according to disease status. Data from each group were compared by *t*-test. All values in the GC group were significantly higher than those in the healthy group. (F, G, H) CEA, CA125, and CA199 positivity rates. The mean Ct values for RNF180 and SFRP2 cancer patients were 26.43±6.20 and 33.63±6.05. Mean Ct values for RNF180 and SFRP2 cancer patients were 26.43±6.20 and 33.63±6.05. ns, p>0.05; *, p≤0.05; **, p≤0.01; ***, p≤0.001.

When comparing the effects of *RNF180* and *SFRP2* with traditional tumor markers (*CEA, CA125,* and *CA199*), significant differences were found between the GC and healthy individual groups for all five genes. The mean Ct values in *RNF180* were 26.43±6.20, 28.42±6.62, and 31.00±7.15 for GC, precancerous gastric lesions, and healthy patients, respectively. The mean Ct values in *SFRP2* were 33.63±6.05, 37.65±4.11, and 37.71±3.83 for GC, precancerous gastric lesions, and healthy patients, respectively. The range of positive Ct values could not be determined because the mean Ct values of the GC group, precancerous gastric lesions group, and healthy group did not differ. Statistics on conventional tumor markers revealed that the positivity rate of each marker in GC did not exceed 25 % (Fig. 1).

### Modeling in the training set

**Fig. 2:**
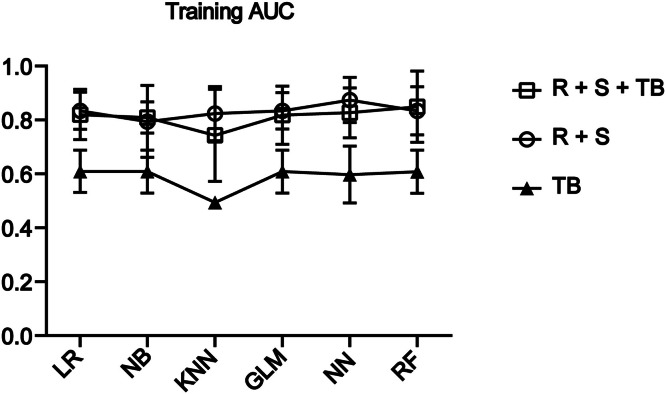
AUC for each model in the training set. The RF model in the RS group and the RS + tumor marker group outperformed the tumor marker group. R + S for RNF180 + SFRP2; TB for tumor biomarkers.

To improve the sensitivity of detecting GC, we included 78 GC patients and 124 healthy individuals in the training set (Supplemental Table S2) and divided them into three groups: *RNF180* and *SFRP2* (RS), a tumor marker group (*CEA, CA125,* and *CA199*), and a group with RS + tumor marker. Six algorithms, including LR, NB, KNN, glmnet, NNET, and RF, were used for analysis to calculate the area under the receiver operating characteristic curve (ROC) (AUC), sensitivity, and specificity for subjects, respectively. The RF algorithm outperformed all other algorithms. The AUC of the RF model for the RS group was 0.839 (95 % CI: 0.727–0.951), with a sensitivity of 60.3 % and specificity of 85.5 %. The AUC of the RF model for the RS + tumor marker group was 0.849 (95 % CI: 0.717–0.981), with a sensitivity of 62.8 % and specificity of 87.1 % (Fig. 2, Table 1).

**Table 1:** presents the AUC, sensitivity, and specificity of the three groups of genes in the training set across the three algorithms.

Model	Genes	AUC	Sensitivity	Specificity
LR	RNF180+SFRP2	0.80	0.54	0.85
NB	RNF180+SFRP2	0.79	0.54	0.84
KNN	RNF180+SFRP2	0.72	0.54	0.79
GLM	RNF180+SFRP2	0.80	0.50	0.85
NN	RNF180+SFRP2	0.83	0.67	0.87
RF	RNF180+SFRP2	0.84	0.60	0.86
LR	TB	0.61	0.24	0.98
NB	TB	0.61	0.09	1
KNN	TB	0.50	0.99	0
GLM	TB	0.61	0.20	0.98
NN	TB	0.60	0.23	0.98
RF	TB	0.61	0.23	0.98
LR	R + S +TB	0.82	0.56	0.81
NB	R + S +TB	0.81	0.29	0.98
KNN	R + S +TB	0.73	0.52	0.81
GLM	R + S +TB	0.82	0.55	0.86
NN	R + S +TB	0.83	0.62	0.86
RF	R + S +TB	0.85	0.62	0.87

### Validating the model in the validation set

**Fig. 3:**
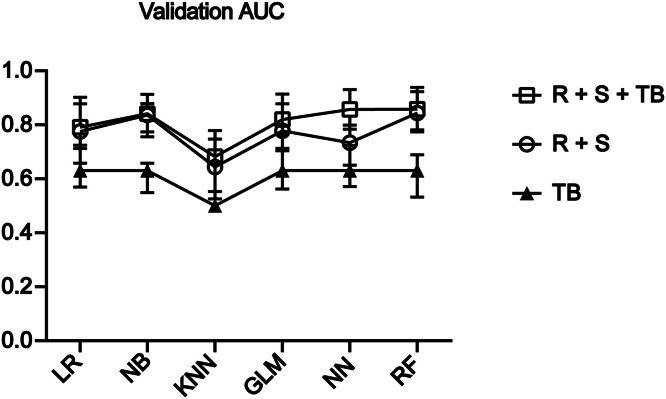
AUC for each model in the validation set. The RF model was superior in the RS and R + S + tumor biomarkers groups.

To validate the model performance in the training set, we compared samples from 101 patients as the validation set. This set contained 60 patients with gastric tumor lesions (26 with GC and 34 with precancerous gastric lesions) and 41 healthy controls (Supplemental Table S3). The validation set was grouped identically to the training set and analyzed using the same algorithm. The results were consistent with the training set, and the RF algorithm outperformed the other algorithms. The AUC of the RF model for the RS group was 0.844 (95 % CI: 0.774–0.923), with a sensitivity of 87.8 % and specificity of 69.2 %. The AUC of the RF model for the RS + tumor marker group was 0.858 (95 % CI: 0.781–0.939), with a sensitivity of 85.4 % and specificity of 76.9 % (Fig. 3, Table 2).

**Table 2:** presents the AUC, sensitivity, and specificity of the three groups of genes in the validation set across the three algorithms.

Model	Genes	AUC	Sensitivity	Specificity
LR	RNF180+SFRP2	0.78	0.81	0.58
NB	RNF180+SFRP2	0.84	0.78	0.77
KNN	RNF180+SFRP2	0.64	0.63	0.65
GLM	RNF180+SFRP2	0.78	0.81	0.62
NN	RNF180+SFRP2	0.73	0.66	0.65
RF	RNF180+SFRP2	0.84	0.88	0.69
LR	TB	0.63	0.95	0.31
NB	TB	0.63	1	0.12
KNN	TB	0.5	0	1
GLM	TB	0.63	0.95	0.27
NN	TB	0.63	0.95	0.31
RF	TB	0.63	0.95	0.31
LR	R + S +TB	0.79	0.80	0.65
NB	R + S +TB	0.84	0.95	0.42
KNN	R + S +TB	0.68	0.63	0.73
GLM	R + S +TB	0.82	0.78	0.62
NN	R + S +TB	0.86	0.73	0.89
RF	R + S +TB	0.86	0.85	0.77

### Comparison of GC prediction ability between the RS R + S and RS + tumor marker groups

**Fig. 4:**
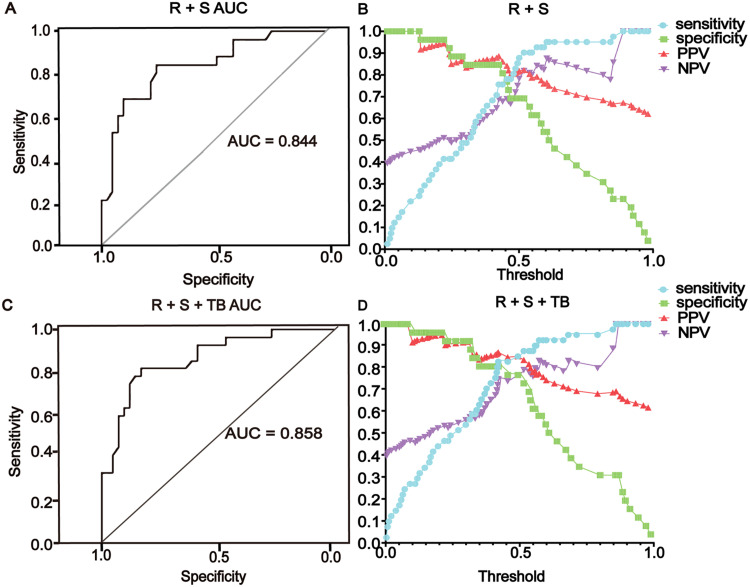
The RF model was chosen to analyze the validation set. (A, B) The AUC, sensitivity, specificity, positive predictive value, and negative predictive value were analyzed using the RS method. The AUC was 0.844. At a threshold of 0.425, the sensitivity was 75.6 %, specificity was 84.6 %, positive predictive value was 88.6 %, and negative predictive value was 68.8 %. (C, D) The AUC, sensitivity, specificity, positive predictive value, and negative predictive value were analyzed using two genes combined with tumor markers. The AUC was 0.858. At a threshold of 0.43, the sensitivity was 82.9 %, specificity was 80.8 %, positive predictive value was 87.2 %, and negative predictive value was 75 %. PPV, positive predictive value; NPV, negative predictive value.

To compare the predictive ability of the RS group and the RS + tumor marker group in the process of GC-assisted diagnosis, the validation set was analyzed using the RF model to assess the AUC, sensitivity, specificity, PPV, NPV, and positive detection rate (PDR). The AUC for the R + S set was 0.844, and when the threshold was 0.425, the sensitivity, specificity, PPV, and NPV were 75.6 %, 84.6 %, 88.6 %, and 68.8 %, respectively. The PDRs of early GC with RF and RS were 80.8 %, and that of advanced GC was 96 % (Supplemental Table S4). The AUC for the RS + tumor marker group was 0.858. When the threshold was 0.43, the sensitivity, specificity, and PPV were 82.9 %, 80.8 %, and 87.2 %, respectively, and the NPV was 75 % (Fig. 4). The positive detection rate of early GC with RF and RS + tumor markers was 80.1 %, and that of advanced GC was 98.7 % (Supplemental Table S5).

### RNF180 and SFRP2 are hypermethylated in gastric cancer

Cellular experiments were conducted to validate the methylation levels of RNF180 and SFRP2 in highly differentiated (HGC-27), undifferentiated (MKN-7), and gastric mucosa cell line (GES-1). The results demonstrated that the DNA methylation levels of RNF180 and SFRP2 were highest in the HGC-27 cell line and lowest in GES-1 cell line (Fig. S1A). The opposite results were observed in RNF180 and SFRP2mRNA levels (Fig. S1B). Furthermore, in Fig. S1C, knockdown of RNF180 or SFRP2 in the HGC-27 cell line enhanced the proliferation of gastric cancer cells.

## Discussion

DNA methylation refers to the methylation of the 5′ carbon atom of cytosine in cytosine-guanine dinucleotides (CpG) to form 5-methylcytosine [47]. It is the most studied form of epigenetic modification. Aberrant promoter methylation inactivates tumor-associated genes, leading to gastric carcinogenesis. It has been shown that RNF180 and SFRP2 are hypermethylated in gastric cancer [48,49], and our study supports this finding. Typically, DNA methylation occurs early in cancer and can be detected in some precancerous lesions [50]. Therefore, methylation of such genes can be used as a biomarker for GC detection. In this study, we selected *RNF180* and *SFRP2* genes from GC patients, patients with precancerous gastric lesions, and healthy individuals, and compared and co-analyzed them with conventional tumor markers (*CEA, CA199,* and *CA125*). The results showed that the combined detection of *RNF180* and *SFRP2* genes outperformed traditional tumor markers in GC diagnosis, with a sensitivity of 85.4 % and specificity of 76.9 %. This suggests that the combination of *RNF180* and *SFRP2* genes improves the diagnostic accuracy of early detection of GC. Our study confirmed the diagnostic value of RNF180 and SFRP2 in GC, indicating that their combination can serve as a biomarker, enhancing the accuracy of gastric cancer diagnosis.

Data from the National Cancer Center (https://seer.cancer.gov/statfacts/html/stomach.html/, 2024 (accessed 8 July 2024)) showed that the 5-year survival rate of early GC was as high as 75.4 %, while the 5-year survival rates of proximal and distal metastatic GC were as low as 35.8 % and 7.0 %, respectively. Early diagnosis of GC is crucial for improving the survival rate. Gastroscopy is the gold standard for diagnosing GC, but it is invasive and has poor compliance. Although traditional tumor markers such as *CEA, CA19–9* and *CA125* are commonly used for GC diagnosis, they have low sensitivity and specificity. Researchers reviewed the clinical significance of serum tumor markers in GC and found that the overall PDR of *CEA* was 24.0 %, and that of CA-199 was 27.0 %[51]. It was reported that *CEA, CA125* and *CA19–9* (PDRs) in GC were 8.3 %, 22.7 %, and 31.1 %, respectively[52]. Our findings indicated that the positive detection rates (PDRs) of *CEA, CA199* and *CA125* were no more than 25 %, consistent with the above data.

Given the heterogeneity of GC, adequate screening sensitivity cannot typically be achieved using a single biomarker [53]. Therefore, current clinical practice focuses on exploring the possibility of combining multiple genes for the diagnosis of GC. It was reported that the AUC, sensitivity, and specificity of SEPTIN9 and RNF180 alone for the diagnosis of GC were insufficient, but these values improved when using a combination of SEPTIN9 and RNF180 [39]. Researchers investigated the diagnostic potential of RNF180 and SFRP2 in detecting GC by analyzing plasma samples for their methylation; while the sensitivity and specificity of RNF180 were 57.89 % and 23.81 %, respectively, and the sensitivity and specificity of SFRP2 were 71.93 % and 42.86 %, respectively, the combined diagnostic approach of RNF180 and SFRP2 for GC diagnosis had higher sensitivity (85.96 %) and specificity (47.62 %)[46].

Consistent with these results, we found that the combined detection of RNF180 and SFRP2 (AUC=0.84) outperformed that of any single tumor marker. We also observed that the performance of RNF180 and SFRP2 combined with traditional tumor markers to predict GC (AUC=0.858) was slightly better than that of the combination of the RNF180 and SFRP2 alone (AUC=0.844). We developed six models for analysis and comparison, identifying the random forest (RF) model as the most effective. This study utilized blood collection, which offers the advantage of being non-invasive. If utilized alongside gastroscopy for diagnosing GC, this blood collection test has the potential to serve as an effective diagnostic tool. Currently, no systematic studies have examined the combination of RNF180 and SFRP2 for gastric cancer diagnosis, nor has the effect of different models on sensitivity been investigated. In this study, we explored a previously neglected aspect by comprehensively investigating the potential of combining RNF180 with SFRP2 for gastric cancer diagnosis. We divided the subjects into training and validation sets, built six models using the training set, and then validated these models in the validation set to identify the most suitable one for gastric cancer diagnosis. Additionally, this study involves blood sampling, which offers the advantages of being non-invasive and less traumatic. This blood sampling method, when used to complement gastroscopy in diagnosing gastric cancer, could become a highly effective tool. Future studies should further explore the combination of RNF180 and SFRP2 with traditional tumor markers or other biomarkers to enhance the accuracy of gastric cancer diagnosis. Additionally, research could focus on monitoring gastric cancer treatment and prognosis, as well as predicting responses to immunotherapy and targeted therapy.

However, there are some limitations to this study. Firstly, the sample size of the validation set was small, which may have contributed to the slight deviation in results between the validation and training sets. Additionally, since all patients in this study were from Zhejiang Provincial Cancer Hospital, it is unclear whether different geographical regions or racial populations would yield similar results. Secondly, we did not analyze the correlation of the mean Ct values of mRNF180 and mSFRP2 with TNM clinical stage, sex, age, tumor location, tumor size, differentiation, and Lauren type. Finally, the results of this study primarily pertain to the detection of gastric cancer in high-risk populations aged 40 years or older and may not be applicable for tumor screening in the general population. Although the methylation detection of RNF180 and SFRP2 shows potential for diagnosing gastric cancer, further technical optimization is necessary to ensure the reproducibility and stability of the assay.

In summary, plasma mRNF180 and mSFRP2 outperformed CEA, CA19–9, and CA125, and they can be regarded as useful and non-invasive biomarkers for the diagnosis of GC. However, the combination of RNF180 and SFRP2 with CEA, CA19–9, and CA125 was found to be more effective. In addition, the RF model has potential clinical applications in assisting with the diagnosis of GC, which could improve patient outcomes.

## Funding

This work was supported by 10.13039/100022963Key Research Development Program of Zhejiang (2022C03015), National Health Commission Research Foundation (WKJ-ZJ-2305), Natural Science Foundation of Zhejiang Province of China (Q23H220002), and Virtue Diagnostics Co., Ltd.

## CRediT authorship contribution statement

**Zhihao Dai:** Writing – original draft, Conceptualization. **Jin Jiang:** Writing – original draft, Investigation. **Qianping Chen:** Writing – original draft, Investigation. **Minghua Bai:** Conceptualization. **Quanquan Sun:** Conceptualization. **Yanru Feng:** Conceptualization. **Dong Liu:** Conceptualization. **Dong Wang:** Conceptualization. **Tong Zhang:** Data curation. **Liang Han:** Data curation. **Litheng Ng:** Data curation. **Jun Zheng:** Data curation. **Hao Zou:** Data curation. **Wei Mao:** Writing – review & editing, Supervision. **Ji Zhu:** Writing – review & editing, Supervision.

## Declaration of competing interest

The authors declare the following financial interests/personal relationships which may be considered as potential competing interests:

Ji Zhu reports financial support was provided by Department of Science and Technology of Zhejiang Province. Ji Zhu reports financial support was provided by Health Development Research Center of the National Health Commission. Wei Mao reports financial support was provided by Natural Science Foundation of Zhejiang Province of China. Qianping Chen reports financial support was provided by Natural Science Foundation of Zhejiang Province of China. The coauthors, Liang Han, Tong Zhang, Litheng Ng, Jun Zheng and Hao Zou are current employees of Virtue Diagnostics Co., Ltd. If there are other authors, they declare that they have no known competing financial interests or personal relationships that could have appeared to influence the work reported in this paper.

## Appendix Supplementary materials

Image, application 1

Image, application 2

Image, application 3

Image, application 4

Image, application 5

Image, application 6
