# Supplementary figures and images for: Combining methylated RNF180 and SFRP2 plasma biomarkers for noninvasive diagnosis of gastric cancer
Source: Transl Oncol. 2024 Nov 13;51:102190. doi: 10.1016/j.tranon.2024.102190 (PMC11600768; doi:10.1016/j.tranon.2024.102190)

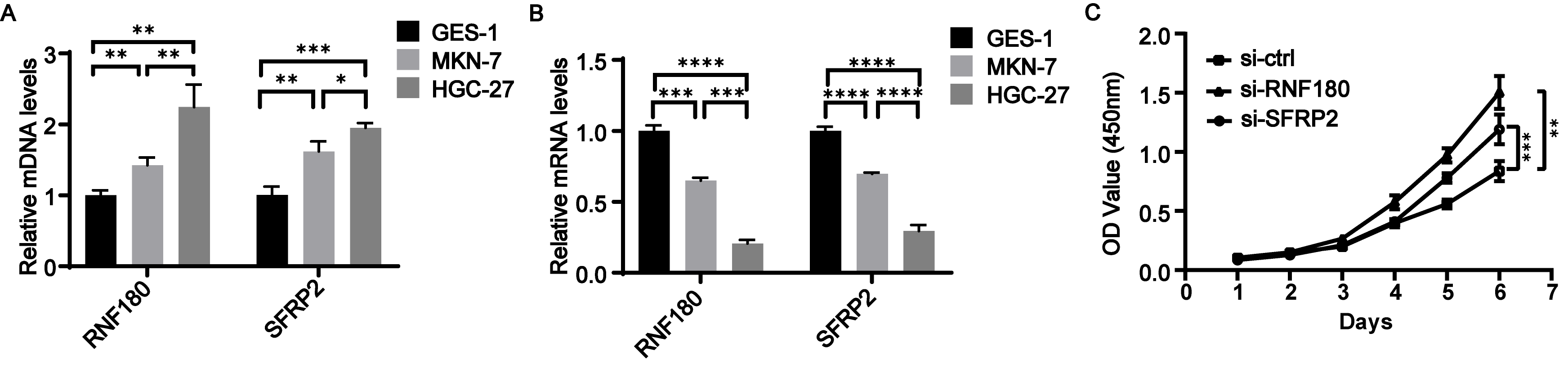

Supplement: Supplementary file 1 [file mmc1.zip › Supplemental Fig 1.tif]

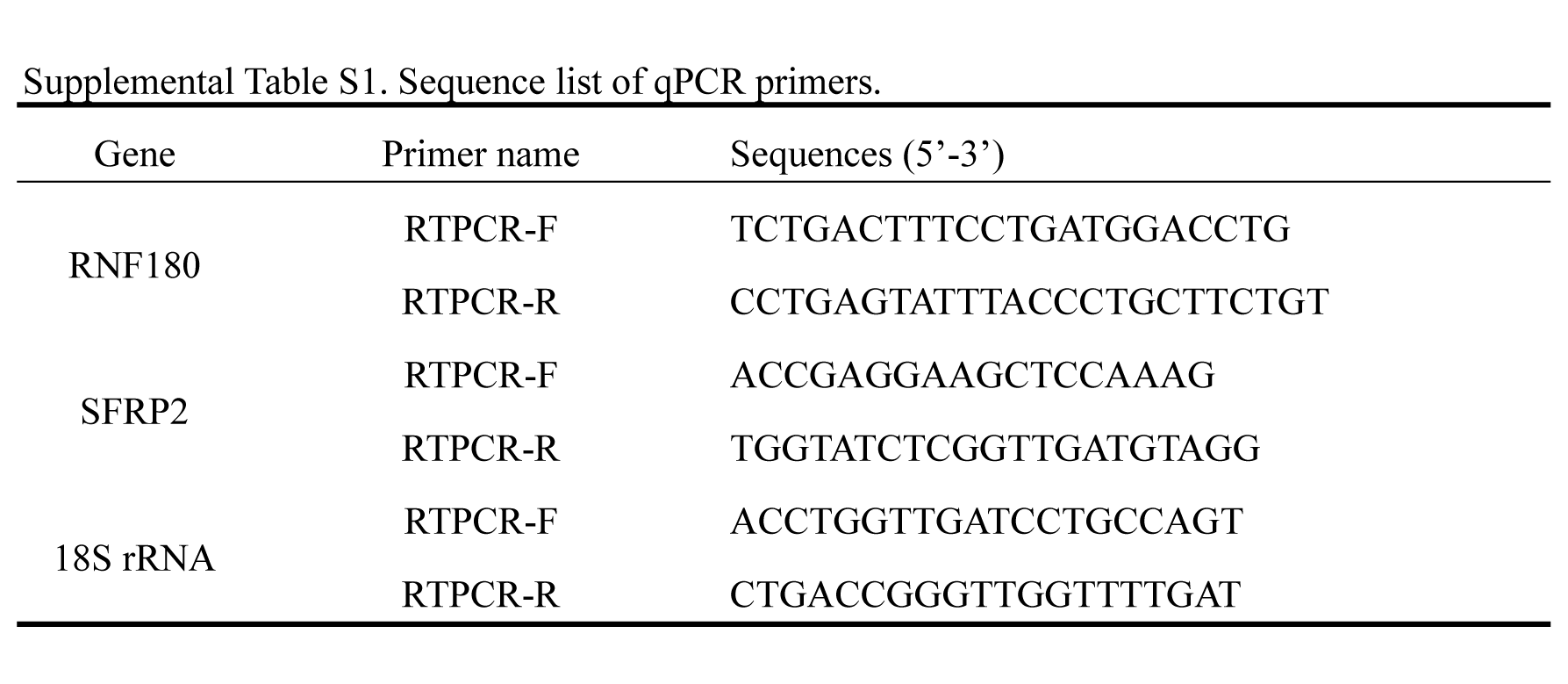

Supplement: Supplementary file 2 [file mmc2.zip › Supplemental Table S1.tif]

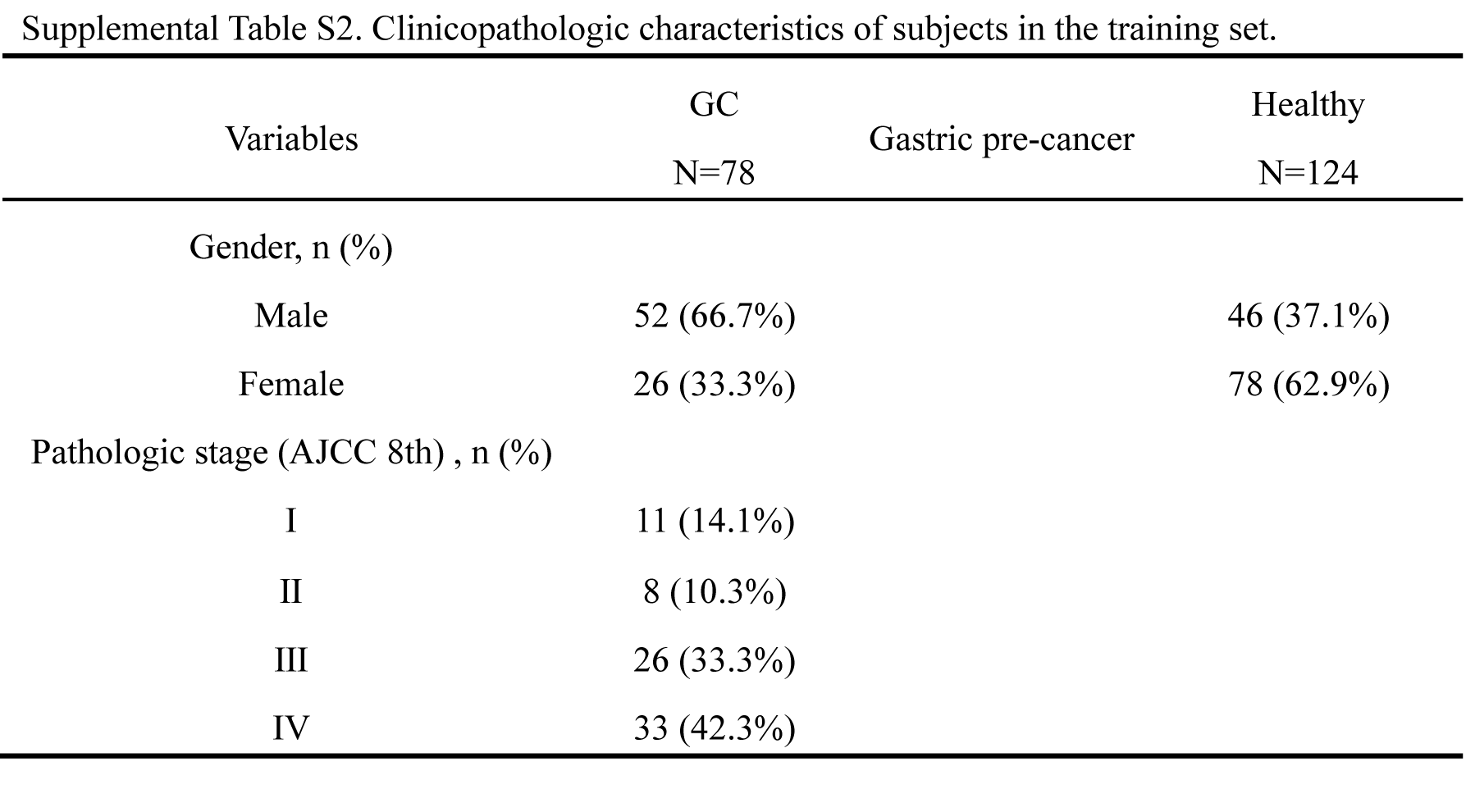

Supplement: Supplementary file 3 [file mmc3.zip › Supplemental Table S2.tif]

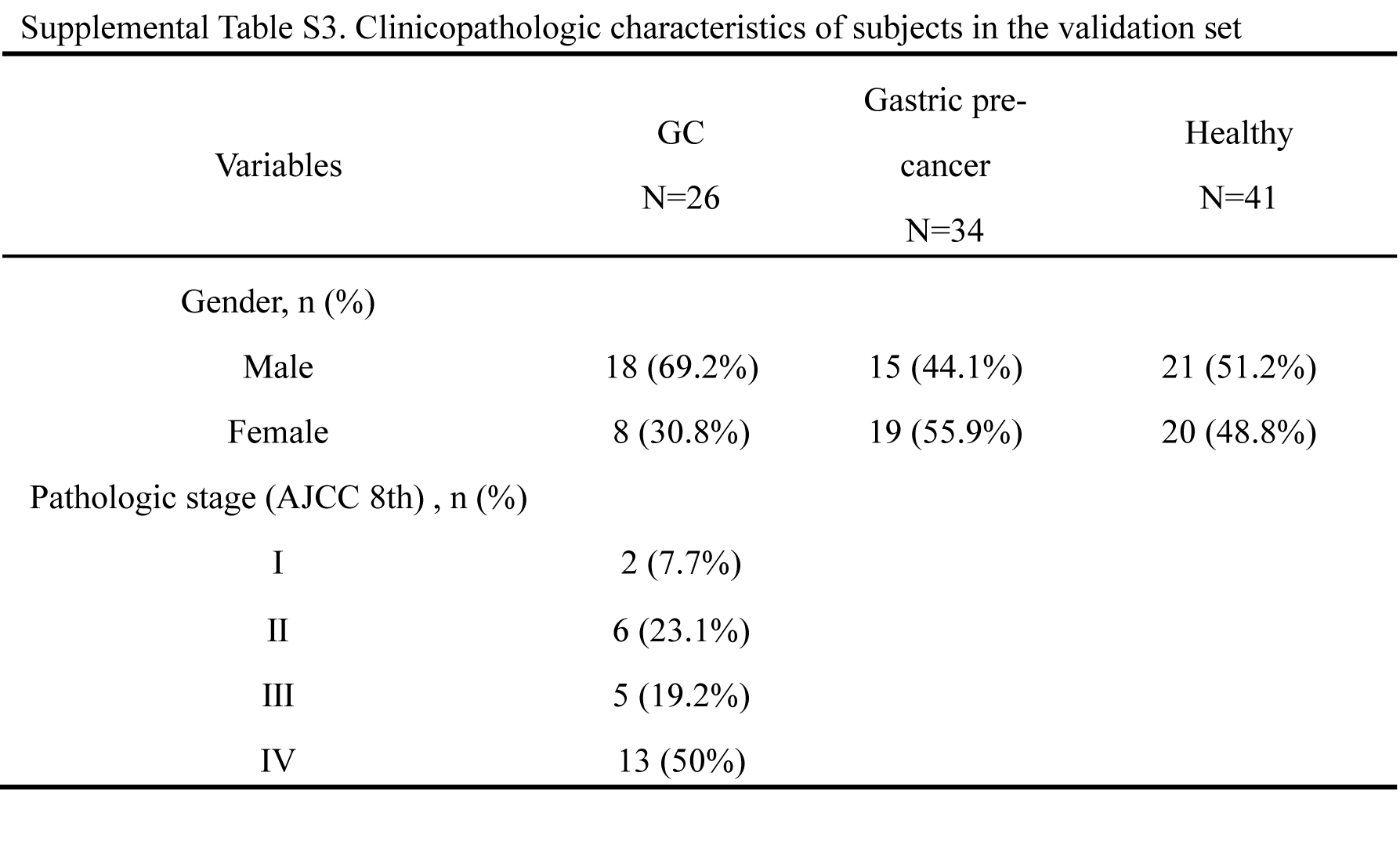

Supplement: Supplementary file 4 [file mmc4.zip › Supplemental Table S3.tif]

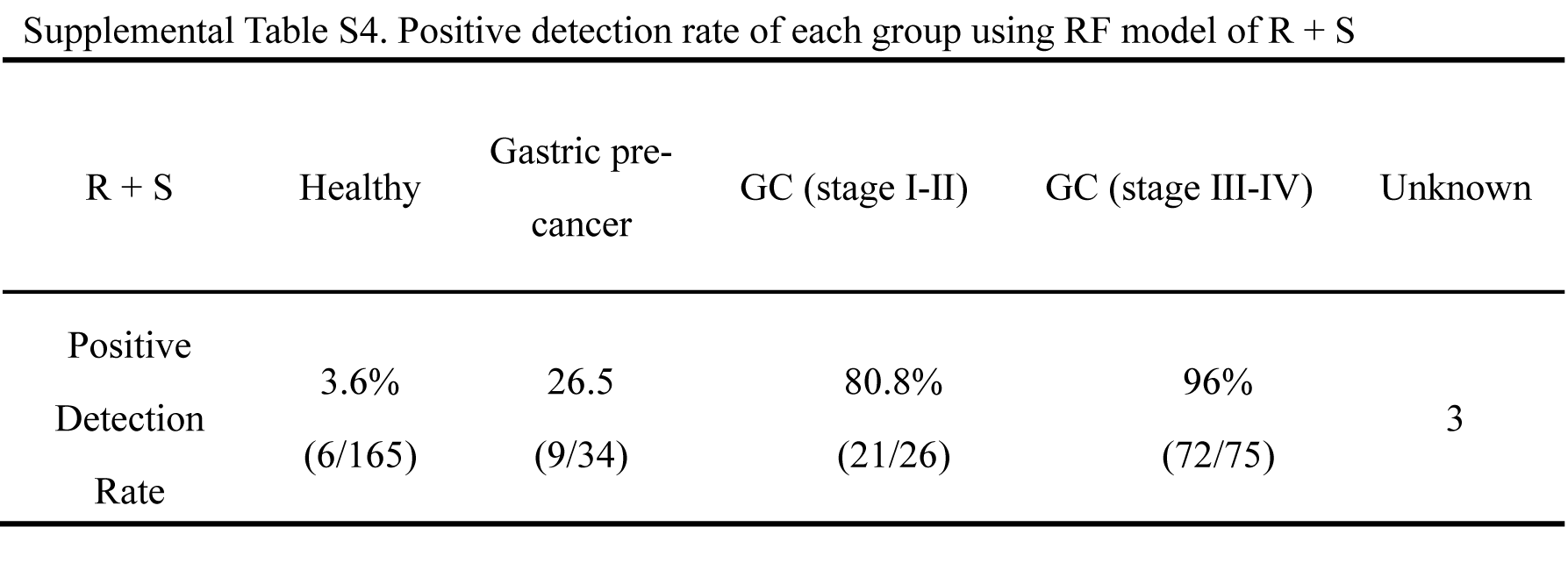

Supplement: Supplementary file 5 [file mmc5.zip › Supplemental Table S4.tif]

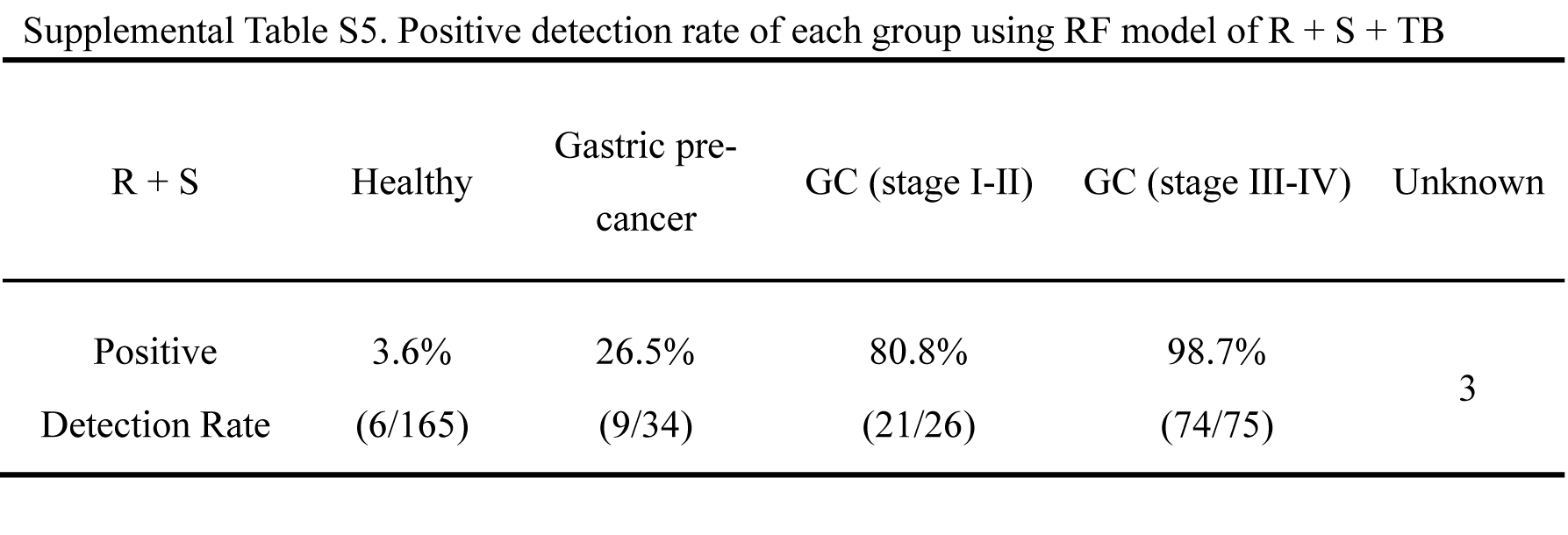

Supplement: Supplementary file 6 [file mmc6.zip › Supplemental Table S5.tif]
